# UK recommendations for chimerism testing and monitoring following allogeneic haematopoietic stem cell transplantation (HSCT): Best practice consensus guidelines from the British Society for Blood and Marrow Transplant and Cellular Therapies (BSBMTCT), NHS England Genomic Laboratory Hub (GLH) Haematological Malignancies Working Group, UK Cancer Genetics Group (UKCGG) and the UK National External Quality Assessment Service for Leucocyte Immunophenotyping (UK NEQAS LI)

**DOI:** 10.1111/bjh.70061

**Published:** 2025-09-09

**Authors:** Andrew Clark, Hazel Clouston, Kanchan Rao, Najeem Folarin, Josu De la Fuente, Angela Hamblin, Eduardo Olavarria, Debbie Richardson, Polly Talley, Victoria Potter, Justin Loke, Terri McVeigh, John Snowden

**Affiliations:** ^1^ Department of Haematology Queen Elizabeth University Hospital Glasgow UK; ^2^ UK NEQAS for Leucocyte Immunophenotyping Sheffield Teaching Hospitals NHS Foundation Trust Sheffield UK; ^3^ Department of Haematology Great Ormond Street Hospital London UK; ^4^ Synnovis Molecular Biology Laboratory Guys and St Thomas' Hospitals NHS Trust London UK; ^5^ Department of Paediatric Haematolgy Imperial College London UK; ^6^ Central and South Genomic Laboratory Hub Oxford University Hospitals NHS Foundation Oxford UK; ^7^ Department of Haematology Imperial College London UK; ^8^ Department of Haematology Southampton University Hospitals Trust Southampton UK; ^9^ Leeds Cancer Institute, Leeds Teaching Hospitals NHS Trust Leeds UK; ^10^ Department of Haematology Kings College Hospital NHS Foundation Trust London UK; ^11^ Centre for Haematology University Hospital Birmingham NHS Foundation Trust Birmingham UK; ^12^ The Institute of Cancer Research London UK; ^13^ Department of Haematology Sheffield Teaching Hospitals NHS Foundation Trust Sheffield UK

**Keywords:** allogeneic stem cell transplant, BMT, chimerism, DLI, PBSCT

## Abstract

In allogeneic haematopoietic stem cell transplantation (HSCT), important clinical decisions depend upon assessment of chimerism, including immunosuppressant dosing and donor lymphocyte infusions (DLI), which in turn can have major impacts on disease control, graft‐versus‐host disease (GVHD), immunity and ultimately patient survival. There is a complex range of clinical and laboratory procedural considerations including methodology of testing, types of cell subset selection, frequency of testing, urgency of turnaround times (TATs), interplay with measurable residual disease (MRD) monitoring and duration of testing post‐transplant. These aspects are routinely adapted according to disease indication, patient characteristics, donor source and intensity of transplant technique. To encourage the harmonisation of clinical and laboratory practice in the United Kingdom, we held a national workshop meeting to bring together key stakeholders to review the current literature with a view to producing a state‐of‐the‐art position paper. Here, we present best practice consensus recommendations and identify key areas for future audit and research from the UK Cancer Genetics Group (UKCGG), NHS England Genomic Laboratory Hub (GLH) Haematological Oncology Malignancies Working Group, UK National External Quality Assessment Service for Leucocyte Immunophenotyping (UK NEQAS LI) and the British Society of Blood and Marrow Transplantation and Cellular Therapy (BSBMTCT).

## INTRODUCTION

Allogeneic haemopoietic stem cell transplantation (HSCT) is a potentially curative treatment for both haematological malignancies and non‐malignant diseases including bone marrow failure syndromes, haemoglobinopathies, congenital immunodeficiencies, autoimmune diseases and metabolic disorders.^1^ During the transplant course, it has become increasingly important to be able to track multilineage engraftment and monitor the relative contribution from host or donor stem cells to post‐transplant lympho‐haemopoiesis. The aims are to prevent rejection, unwanted autologous reconstitution or relapse of malignant disease over time, in what is a complex, dynamic process. Chimerism analysis performs these functions utilising molecular differences between host and donor cells. It informs many aspects of post‐transplant management acting as a trigger for interventions including modulation of immunosuppression, CD34+ selected ‘top‐ups’, second transplants and pre‐emptive donor lymphocyte infusions (‘preDLI’).

Laboratory testing must be robust, reliable and responsive to clinical need to facilitate decision‐making according to the tempo of the underlying disease. A collegiate approach to the choice of tests, the schedule of testing, the reporting of results with clear reference to quality assurance parameters and their interpretation is important. Further standardisation should allow the effect of interventions to be systematically studied through clear audit and research strategies. Although newer technologies offer the exciting prospect of more sensitive and detailed dissection of these processes and may more effectively inform management decisions, careful thought is required when introducing these platforms. In the clinic, the optimal testing strategies and thresholds for intervention need to be identified to allow national prospective audit of clinical outcomes. Recommendations should account for the spectrum of disease indications in adult and paediatric practice as well as the intensity of transplant conditioning and other procedural aspects.

The wide variation in clinical and laboratory practices in this area highlights the need for evidence‐based, expert‐led consensus guidelines within UK NHS provision, with potential relevance to health services in other countries. To achieve these goals, representatives from UK transplant programmes (adult and paediatric) and associated laboratories participated in an online workshop followed by an iterative review process.

## METHODS

This initiative was led by the British Society for Blood and Marrow Transplantation and Cellular Therapy (BSBMTCT) and included representation from the UK Cancer Genetics Group (UK CGG), a constituent group of the British Society of Genomic Medicine, NHS England Genomics Haematological Malignancies Working Group and UK NEQAS Leucocyte Immunophenotyping (UK NEQAS LI). First, a virtual workshop was held in March 2024 dedicated to discussing the place of chimerism testing in allogeneic HSCT—including laboratory testing considerations, the necessity of external quality assurance (EQA) and practical applications of chimerism testing in adult and paediatric practice. This involved key experts in HSCT, Clinical Genetics and Genetic testing laboratories with a UK NHS focus, but with relevance to HSCT programmes and health services in other countries. The methodology for the transplant‐specific workshop was similar to that employed previously.^2^, ^3^ The draft consensus document was then circulated to UK transplant directors, genetic laboratory participants and key stakeholders for additional comments which were included in the final manuscript.

### Development of consensus recommendations

The final consensus recommendations were developed using:

1. In‐meeting polling to address targeted questions. Using this approach, all meeting attendees were involved in reaching consensus in real time.
2. In‐meeting discussion following polling. These discussions were informed by state‐of‐the‐art presentations on the day and subsequently collated. They complemented the in‐meeting polling.
3. Further recommendations were produced with a view to harmonising practice in line with the targeted consensus statements and/or refining them in the light of published evidence.
4. Comments on the meeting manuscript from UK transplant directors, genetic or H&I laboratory participants and key stakeholders were used to modify the final consensus.

Individual recommendations carry superscript numbering to reflect these sources. Full in‐meeting polling results are listed in Tables 1, 2, 3.

**TABLE 1:** Statements on which consensus ≥80% agreement was reached.

1. Peripheral blood is the specimen of choice for chimerism analysis in most circumstances (*n* = 55 respondents) (Agree/strongly agree: 88%, disagree: 7%, neutral: 5%)
2. Chimerism analysis of bone marrow may be indicated in certain circumstances such as mixed whole blood or myeloid chimerism (*n* = 50 respondents) (Agree/strongly agree: 80%, neutral: 12%, disagree: 8%)
3. Analysis of chimerism using FISH techniques is rarely indicated (*n* = 46 respondents) (Agree/strongly agree: 83%, neutral: 13%, disagree: 4%)
4. STR techniques currently represent standard of care for chimerism analysis in the United Kingdom as they are rapid, reliable, accurate and reproducible despite limited sensitivity of 1%–5% (*n* = 61 respondents) (Agree/strongly agree: 93%, neutral: 5%, disagree: 2%)
5. Future research should be targeted to assessment of the utility of more sensitive molecular tests in predicting relapse early post‐transplant (*n* = 45 respondents) (Agree/strongly agree: 93%, neutral: 2%, disagree: 5%)
6. Chimerism analysis and MRD monitoring using molecular methods are important complementary monitoring modalities post‐allogeneic stem cell transplant and should be monitored in tandem (*n* = 60 respondents) (Agree/strongly agree: 98%, neutral: 2%, disagree: 0%)
7. Laboratory reports must include the limit of detection of the assay utilised (*n* = 53 respondents) (Agree/strongly agree: 89%, neutral: 8%, disagree: 4%)
8. Cell separation techniques and lineage‐specific enrichment may be carried out using magnetic beads or flow cytometry‐based techniques (*n* = 42 respondents) (Agree/strongly agree: 94%, neutral: 6%, disagree: 0%)
9. Purity of the lineage‐specific subsets should form part of the chimerism report (*n* = 47 respondents) (Agree/strongly agree: 89% neutral: 9%, disagree: 2%)
10. The turnaround time for chimerism testing for routine testing should be <2 weeks (*n* = 52 respondents) (Agree/strongly agree: 88%, neutral: 6%, disagree: 6%)
11. The turnaround time for chimerism testing for urgent testing should be <5 working days (*n* = 49 respondents) (Agree/strongly agree: 83%, neutral: 11%, disagree: 6%)
12. It is essential to participate in an EQA scheme for chimerism testing to promote standardisation, allow comparison of techniques/kits and identify factors associated with satisfactory/unsatisfactory performance (*n* = 57 respondents) (Agree/strongly agree: 99%, neutral: 1%, disagree: 0%)
13. The EQA scheme should ideally include cell lineage selection when technical issues allowing this to be possible are resolved (*n* = 47 respondents) (Agree/strongly agree: 94%; neutral: 6%; disagree: 0%)
14. Depending on transplant indication, the schedule and type of chimerism testing (rate/duration of testing, analysis of cell subsets) should be standardised considering also intensity of transplant conditioning (*n* = 48 respondents) (Agree/strongly agree: 81%, neutral: 13%, disagree: 6%)
15. Whole blood chimerism should be performed at day 30 using STR technology and can yield useful information regarding engraftment or incipient relapse (*n* = 46 respondents) (Agree/strongly agree: 80%, neutral: 12%, disagree: 8%)
16. Assessment of myeloid or whole blood chimerism at day 30 using more sensitive techniques may improve detection of patients at risk of relapse and should be assessed in clinical trials (*n* = 48 respondents) (Agree/strongly agree: 80%, neutral: 20%, disagree: 0%)

Abbreviations: ALD, adrenoleukodystrophy; CGD, chronic granulmatous disease; DBA, diamond blackfan anaemia; FA, fanconi anaemia; HLH, haemophagocytic lymphohistiocytosis; SCID, severe combined immunodeficiency; SCN, severe congeital neutropenia; SDS, schwachman diamond syndrome; WAS, wiskott‐aldrich syndrome.

## RESULTS

### Participants

A full list of participants is given in Appendix. The meeting demographics included wide representation from all stakeholders in this area. There were 41 clinical geneticists, nine paediatric consultant haematologists, 21 adult haematologists/BMT consultants, four clinical nurse specialists and three participants did not disclose full affiliations. Sixty‐eight participants participated in the in‐meeting polling.

### In‐meeting polls

Consensus over 80% was achieved on 16 statements (Table 1). One statement did not have enough respondents to be accepted but was strongly supported. A further seven statements were agreed by the majority, but this fell short of an 80% agreement (Table 2). In four of these, less than 15% of respondents disagreed, but a proportion did not know and voted ‘neutral’. Two statements were strongly rejected (Table 3).

**TABLE 2:** Statements on which the majority of participants agreed (60%–80% ‘agreement/strong agreement’ or fewer than 40 respondents).

1. In malignant diseases, mixed T‐cell chimerism <90% requires clinical intervention with tapering of immunosuppression ± DLI (*n* = 20 respondents) (Agree/strongly agree: 85%, neutral: 4%, disagree: 11%)
2. Chimerism analysis using more sensitive molecular techniques such as qPCR, dPCR and NGS does not yet offer enough additional benefit to become standard of care (*n* = 61 respondents) (Agree/strongly agree: 65%, neutral: 20%, disagree: 15%)
3. Reports should include the minimum value of % donor chimerism compatible with full donor chimerism for any given assay (*n* = 51 respondents) (Agree/strongly agree: 69%, neutral: 17%, disagree: 14%)
4. In malignant diseases in adults, assessment of split chimerism in both the T‐cell fraction (CD3) and myeloid fraction (CD15) at day 60, day 100 and 3 monthly to 1 or 2 years should be regarded as a standard of care (*n* = 41 respondents) (Agree/strongly agree: 65%%, neutral: 17%, disagree: 18%)
5. In malignant diseases in children, assessment of split chimerism in both the T‐cell fraction (CD3) and myeloid fraction (CD15) at day 60, day 100 and 3 monthly to 1 or 2 years should be regarded as a standard of care (*n* = 29 respondents) (Agree/strongly agree: 76%, neutral: 15%, disagree: 9%)
6. For patients undergoing transplant for non‐malignant conditions (predominantly in children) where mixed chimerism is observed, long‐term follow‐up to 5 years is needed to assess for clonal evolution or other disease manifestations (*n* = 30 respondents) (Agree/strongly agree: 70%, neutral: 19%, disagree: 11%)
7. Patients undergoing transplant for malignant disease demonstrating 100% donor chimerism in all lineages for 2 years do not need any further chimerism follow‐up (*n* = 30 respondents) (Strongly agree/agree: 63%, neutral: 9%, disagree: 28%)
8. In malignant diseases, mixed T‐cell chimerism >90% is concerning but given the sensitivity of current techniques requires only ongoing monitoring without other intervention (*n* = 33 respondents) (Strongly agree/agree: 60%, neutral: 3%, disagree: 36%)

**TABLE 3:** Statements on which no consensus was reached and no majority opinion was recorded.

1. Mixed myeloid chimerism at any time point post‐transplant is always a concern and should trigger further investigations (*n* = 47 respondents) (Strongly agree/agree: 36%, neutral: 20%, disagree: 46%)
2. MRD techniques are often more sensitive than assessment of chimerism using STR platforms and in proven relapse will often render chimerism analysis unnecessary (*n* = 49 respondents) (Strongly agree/agree: 38%, neutral: 14%, disagree: 48%)

## CONSENSUS RECOMMENDATIONS

### Current chimerism analysis and emerging technologies

The optimal testing strategy used in chimerism monitoring needs to be informative, sensitive, quantitatively accurate, reproducible and cost‐effective.^4^, ^5^, ^6^ Relevant technical guidelines have previously been published by members of this group.^7^ To help establish the best choice of technique used for chimerism monitoring, the meeting reviewed data collected by UK NEQAS LI within their Post‐Stem Cell Transplant Chimerism Monitoring EQA programme. Approximately two‐thirds (66%) of participants, including all those based in the United Kingdom, currently assess chimerism via analysis of short tandem repeats (STRs). This is a well‐established, rapid and robust approach with a limit of detection in the region of 1%–5%. The number of participants adopting alternative technologies, including real‐time quantitative polymerase chain reaction (PCR) (qPCR, 14%), next‐generation sequencing (NGS, 13%) and digital PCR (dPCR, 7%), is increasing globally, and the greater sensitivity of these approaches, particularly qPCR and dPCR, offers the potential to investigate microchimerism and intervene earlier.^8^, ^9^, ^10^

There are some practical difficulties in introducing more sensitive molecular testing. In the laboratory, these include a reduced ability to accurately quantify significant mixed chimerism (qPCR), increased costs and potentially limited access to specialist equipment (NGS and dPCR), as well as increased processing time (NGS). While in the clinic, the interpretation of these results is challenging. So far, there is a paucity of published data establishing meaningful levels of mixed chimerism that predict relapse or survival parameters associated with the degree of microchimerism that these methods can detect. Similarly, it is not known whether earlier intervention using such highly sensitive techniques influences outcomes. Currently, the well‐established STR approach provides a cost‐effective, pragmatic compromise that largely continues to inform clinical decisions across the United Kingdom. The limitations associated with more sensitive molecular techniques in relation to laboratory testing and clinical utility mean these newer technologies have yet to be widely adopted. Robust change control policies and validation strategies with parallel testing of new technologies alongside established tests, especially with regard to defining threshold levels of mixed chimerism that dictate treatment, are strongly suggested. Clinical reports must include the assay's limit of detection (LoD) to allow correct interpretation of the result: for example, there is a fundamental difference in the significance of 98% donor chimerism when generated by an assay with an LoD of 3% versus an assay with an LoD of 0.5%.

There is clinical utility, in certain situations, for examining chimerism in specific lineages, including T cells (CD3+), B cells (CD19+), myeloid lineages (e.g. CD15+) and stem cells (CD34+). For lineage‐specific chimerism, cell separation techniques include the use of immunomagnetic beads and cell sorting using a flow cytometer. Sorting is carried out prior to DNA extraction. UK NEQAS LI data show that, of 15 UK respondents, 13 provide lineage‐specific chimerism. All 13 offered T‐cell chimerism, while seven offered B‐cell chimerism, 11 offered myeloid chimerism and two offered chimerism for any cell type requested by the referring clinician.

Knowing the percentage of the target cell in the selected population from which the DNA was extracted is crucial. Many of the informative cell subsets, for instance CD3+ T lymphocytes following a T‐depleted transplant, are present in very small numbers, especially early post‐transplant, and contamination from other lineages can lead to spurious results. Practice here is variable. Nine laboratories assess the purity of the separated cells (eight by flow cytometry, one by PCR); two laboratories do not provide a purity assessment; and two laboratories stated ‘not applicable’ as the separation is performed in an alternative centre. Of the nine respondents assessing purity, three did not have a minimum purity for reporting. Minimum purities for the other six laboratories range from 60% to 95%.

### External quality assessment in post‐transplant chimerism monitoring

A well‐established external quality assessment programme for chimerism analysis has been provided by UK NEQAS LI since 2008. This programme is accredited by the United Kingdom Accreditation Service (UKAS) and currently has 113 participating centres, of which 20 are in the United Kingdom. Five trials are issued per annum, each requiring assessment of the % donor chimerism in two post‐transplant samples. Trial submissions capture the range of methodologies in use, and performance monitoring is based on robust statistics and *z*‐scores.^11^, ^12^ Participation in EQA is an essential requirement for successful laboratory accreditation by accreditation bodies such as the European Federation for Immunogenetics (EFI) and UKAS. Ideally, chimerism programmes would encompass lineage‐specific chimerism analysis; however, such EQA provision is technically challenging and currently remains under development.

### Recommendations for laboratory assessment of chimerism

- **Peripheral blood is the specimen of choice for chimerism analysis in most circumstances.** ^1^
- **Chimerism analysis of bone marrow may be indicated in certain circumstances such as mixed whole blood or myeloid chimerism.** 1, 2
- **Analysis of chimerism using FISH techniques is rarely indicated unless molecular genetic testing (e.g. STR analysis) is not suitable.** 1, 2
- **STR techniques currently represent standard of care for chimerism analysis in the UK as they are rapid, reliable, accurate and reproducible despite limited sensitivity of 1%–5%.** ^1^
- **Chimerism analysis using more sensitive molecular techniques such as qPCR, dPCR and NGS does not yet offer enough additional benefit to become standard of care.** 1, 2
- **Future research should be targeted to assessment of the utility of more sensitive molecular tests in defining chimerism and predicting relapse early post‐transplant.** 1, 2
- **Cell separation techniques and lineage‐specific enrichment may be carried out using magnetic beads or flow cytometry‐based techniques.** ^1^
- **Repeat testing and confirmation of abnormal results is usually necessary prior to clinical intervention.** ^3^
- **Laboratory reports should include the limit of detection of the assay utilised.** ^1^
- **Reports should include the minimum value of percentage donor chimerism compatible with full donor chimerism for any given assay.** ^1^
- **The turnaround time for chimerism testing for routine testing should be <2 weeks.** ^1^
- **The turnaround time for chimerism testing for urgent testing should be <5 working days.** ^1^
- **Purity of any lineage‐specific subsets should form part of the chimerism report.** ^1^
- **It is essential to participate in an EQA scheme for chimerism testing to promote standardisation, allow comparison of techniques/kits and identify factors associated with satisfactory/unsatisfactory performance.** ^1^
- **The EQA scheme should ideally include cell lineage selection when technical issues allowing this to be possible are resolved.** ^1^

### Post‐transplant chimerism testing, monitoring and clinical utility in adult BMT practice

#### Malignant disease

In adults, most allogeneic transplants are performed for malignant disease where chimerism results, often in tandem with MRD assessments, are used to determine the timing and speed of the withdrawal of immunosuppression (IS) and administration of ‘preDLI’.^13^

The predictive value of mixed chimerism on outcomes may be influenced by the cell subset analysed, intensity of conditioning and T depletion. Myeloablative transplantation is usually associated with sustained full donor chimerism^14^ and falling levels of whole blood donor chimerism of >5% in sequential testing have been associated with a significant increase in relapse in both the T‐replete and T‐depleted settings.^15^, ^16^ In T‐replete reduced intensity conditioned (RIC) transplants, mixed chimerism with <95% donor cells in unfractionated peripheral blood (PB) or a delay in achieving donor T‐cell chimerism have been associated with poorer PFS and increased relapse. While in RIC transplants including anti thymocyte globulin (ATG) in conditioning, mixed T‐cell chimerism of <95% donor at D + 30 and D + 100 has been associated with increased relapse rates (HR = 0.90, *p* < 0.001), reduced relapse‐free (HR = 0.89, *p* < 0.001) and overall survival (HR = 0.94, *p* = 0.01).^17^, ^18^, ^19^, ^20^ If alemtuzumab is used in conditioning, worsening mixed chimerism after cessation of immunosuppression clearly increases the relapse risk^21^ and, while it was initially unclear if stable mixed donor chimerism in the T‐cell lineage constituted a risk factor for relapse, most subsequent studies have suggested that conversion to full donor chimerism, either spontaneously or after DLI, did reduce relapse risk.^13^, ^22^, ^23^, ^24^ Retrospective studies, the largest published by EBMT, have also shown that DLI are an effective treatment for strengthening the GvL effect if mixed chimerism is detected.^25^, ^26^, ^27^, ^28^ The EBMT study included 169 with acute leukaemia who received ‘preDLI’ for mixed chimerism alone. Efficacy of ‘preDLI’ was demonstrated by decreasing MRD/increasing blood counts in 71% and increasing chimerism in 70%. Five‐year OS after ‘preDLI’ for MRD/MC was 51%/68% among responders and 37% among non‐responders.^25^ UK studies have used CD3+ T lymphoid chimerism <95% as a trigger for DLI in T‐depleted RIC transplants with good efficacy and low rates of graft‐versus‐host disease (GVHD), especially if DLI administration is fractionated.^13^, ^22^, ^23^, ^29^

UK adult practice in acute lymphoblastic leukaemia (ALL) is that fit young adults most often receive a myeloablative conditioning regimen including total body irradiation (TBI). In this group, chimerism is usually fully donor in all compartments from early time points^14^, ^15^, ^16^ and any emerging mixed chimerism, especially early post‐transplant is always a cause for concern. Older patients or those with comorbidities routinely receive a T‐depleted RIC regimen as per the UKALL14 trial protocol.^29^ In UKALL14, following fludarabine, melphalan and alemtuzumab (FMA) conditioning multilineage chimerism was assessed in the peripheral blood every 3 months for 2 years. Mixed chimerism <95% donor in T cells at 6 months occurred in half the patients and was managed by escalating donor lymphocyte infusion doses starting at 1 × 10^6^ cells per kg, increasing by half a log every 3 months if there was no GVHD. 90% of these patients, when subsequently assessed, had developed full donor chimerism in the T‐cell lineage at a median of 5.5 months. Using this approach, 4‐year event free survival (EFS) and OS were 46.7% and 54.8% respectively.^29^ The dynamics of mixed T lymphocyte (CD3) chimerism in this trial and response to ‘preDLI’ support routine use of DLI in this context in tandem with IgH/TCR molecular MRD monitoring. If MRD is negative, DLI can usually be delayed until after 6 months.

In AML, younger adults usually receive myeloablative transplants and those aged over 55 or with comorbidities receive RIC conditioning, a large proportion of whom receive T‐depleting serotherapy or PTCy. Mixed T‐cell chimerism is more commonly seen if full‐dose TBI does not form part of the conditioning regimen, and when T‐cell depletion is used. It has been hypothesised to reflect bidirectional tolerance^30^ which might plausibly affect the graft‐versus‐leukaemia effect. UK practice using chimerism to alter management in T‐depleted RIC allografts has been influenced by the FIGARO study. This randomised controlled trial was a prospective analysis of post‐transplant mixed T‐cell chimerism and measurable residual disease (MRD), alongside other peri‐transplant factors affecting outcomes and was designed to compare the relative efficacy of the FLAMSA‐Bu with standard reduced intensity conditioning regimens that employed in vivo T‐cell depletion. Patients who were alive and relapse free were assessed for chimerism and flow cytometric MRD on day +42, months +3/+6/+9/+12. Patients with full donor T‐cell chimerism (FDTC) at 3 months had an improved OS as compared with patients with mixed donor T‐cell chimerism (MDTC) and had a reduced incidence of post‐transplant MRD positivity. In patients with MDTC (months +3 or +6), MRD positivity was associated with a decreased 2‐year OS; in contrast, in the group with FDTC, MRD was infrequent and did not affect the outcome.^31^ This has led to judicious tapering of IS and ‘preDLI’ being advocated for AML patients with MDTC when using flow MRD or in those with AML at high risk of relapse. However, if highly sensitive molecular monitoring is available such as NPM1, then an alternative approach is to use MRD as the trigger for DLI and tolerate mixed chimerism.

Adult patients undergoing an allogeneic HSCT for mature lymphoid malignancies have peripheral blood chimerism routinely monitored, and mixed chimerism in the T‐cell lineage (CD3) is considered informative with DLI being administered for persistent MDTC <95% donor. In this setting, pre‐DLI is often delayed until after 6 months post‐transplant in the absence of disease recurrence.^22^, ^23^, ^24^

In myelofibrosis, finding mixed myeloid or unfractionated chimerism <95% donor or T lymphoid chimerism <90% donor alone does not lead to early ‘preDLI’, but mandates careful evaluation of molecular MRD. Donor lymphocyte infusions given for molecular relapse induced a higher rate of molecular remission (88%) than DLI for haematological relapse (60%), with a corresponding lower incidence of GVHD. In about half of patients, molecular remission can be achieved without causing GVHD.^32^

#### Non‐malignant diseases

In non‐malignant diseases, adult practice closely follows that discussed in detail in the paediatric section. In aplastic anaemia in the United Kingdom, the use of fludarabine, cyclophosphamide and alemtuzumab has become standard practice. Mixed T‐cell chimerism is frequent and persists after cessation of immunosuppression.^33^ The regimen leads to successful treatment of aplastic anaemia with low rates of GvHD and long‐term mixed T‐cell chimerism indicating the establishment of mutual tolerance. No intervention is necessary for stable mixed T lymphoid chimerism. Mixed myeloid chimerism can be associated with recurrent cytopenias or occur with a stable full blood count. If the blood count remains stable, then no intervention for mixed myeloid chimerism is required. Falling myeloid chimerism in the presence of clinically significant cytopenias may be restored by re‐institution of immunosuppressive therapy. If that fails, then second allogeneic HSCT may be required.

In haemoglobinopathies, adult HSCT practice is evolving. Traditionally, when full intensity myeloablative conditioning regimens were used, less monitoring of mixed chimerism was practised. The feasibility and availability of HSCT in adult sickle cell disorders have been transformed by the introduction of significantly reduced intensity conditioning for sibling HSCT using alemtuzumab and low‐dose TBI,^34^, ^35^ which fosters early mixed CD3+ chimerism, and reduced intensity haploidentical HSCT with PTCy,^36^ but these approaches have a risk of secondary graft failure. The early promise of relatively low donor myeloid fraction always being sufficient to correct erythropoiesis^37^ was not sustained in the face of high risk of secondary graft failure and clonal evolution.^38^ Unlike aplastic anaemia, there is intense competition from the host myeloid compartment, and frequent monitoring of chimerism in myeloid and lymphoid lineages is essential to guide the management of immunosuppression in the post‐transplant with the aim to minimise mixed chimerism.^39^

In any allogeneic HSCT, chimerism analysis early after HSCT can be used to separate poor graft function, where a CD34+ ‘top‐up’ may be indicated, and rejection where more aggressive immunosuppressive therapy or a second HSCT will be required.^5^ Whole blood and surprisingly given the degree of lymphopenia often present, CD3 T‐cell chimerism can both be informative.

- **Whole blood chimerism (unfractionated PB/BM) performed at day 30 using STR technology is recommended as it can yield useful information regarding engraftment or incipient relapse.** ^1^
- **In malignant disease, assessment of cell subsets or unfractionated whole blood chimerism at day 30 using more sensitive techniques may improve the detection of patients at risk of relapse and should be assessed in clinical trials.** ^1^, ^2^
- **In malignant disease, mixed myeloid chimerism is always a concern and should trigger further investigation.** ^3^, ^4^
- **Patients undergoing transplant for malignant disease demonstrating full donor chimerism for 2 years from the time point of last intervention do not need any further chimerism follow‐up.** ^1^, ^2^
- **Recommendations for the use of DLI in response to mixed chimerism have recently been published by EBMT and should be referenced in institutional policies.** ^3^
- **In haemoglobinopathies, assessment of whole blood and lineage‐specific chimerism in, for example, T‐cell fraction (CD3), myeloid fraction (CD15) at day 28, 60, 100 and at least 3 monthly to cessation of immunosuppression is standard of care to guide the management of immunosuppression.** ^1^, ^2^
- **In malignant diseases in adults assessment of whole blood and lineage‐specific chimerism in the T‐cell fraction (CD3) and myeloid fraction (CD15) at day 60, 100 and 3 monthly to 2 years is a standard of care.** ^1^, ^2^
- **In malignant diseases in adults, persistent mixed T‐cell chimerism <95% by STR testing or falling donor T cells (>20%) dictates clinical intervention with tapering of immunosuppression ± preDLI.** ^3^, ^4^
- **Chimerism analysis and MRD monitoring using molecular methods are important complementary monitoring modalities post‐allogeneic stem cell transplant and should be monitored in tandem.** ^1^, ^2^

### Post‐transplant chimerism testing, monitoring and clinical utility in children

#### Malignant disease indications

In malignant diseases in children, there is no consensus internationally on whether mixed chimerism post‐HSCT predicts an increased relapse risk although some studies have shown a correlation between serial mixed chimerism and relapse risk.^16^ Most transplants are myeloablative. The withdrawal of IS on the detection of mixed chimerism in any lineage is common practice, but DLI is less frequently used than in adult practice. The co‐relation with MRD is recognised, and the combination of MRD positivity and mixed chimerism may trigger the decision to implement DLI. The practical difficulties in obtaining DLI from paediatric age donors are another reason for the infrequent use of DLI in children. Overall, DLI, if used, is more commonly used after HSCT for AML than ALL.

In ALL, most centres monitor monthly whole blood chimerism in the first 6 months. Lineage‐specific (T and myeloid) chimerism is checked if there is any evidence of mixed unfractionated chimerism. BMA is performed if mixed chimerism is co‐related with MRD. In the United Kingdom, especially since the availability of CAR‐T‐cell therapy, it is uncommon to administer DLI to correct mixed chimerism post‐HSCT for ALL, as significant GVHD post‐DLI could preclude future CAR‐T therapy. Although if a transplant has been performed after failed CAR‐T‐cell therapy, then DLI is a more attractive clinical option. Recently, an increased incidence of early and significant mixed chimerism has been noted after TBI conditioned transplants for ALL in those children who were bridged to transplant with blinatumomab, compared to standard chemotherapy. As of now, with a median follow‐up of 2 years, there does not seem to be an increased incidence of relapse in this group (personal communication K Rao). Withdrawal of IS or DLI did not change chimerism in this group. Most centres monitor chimerism 4–12 weekly in the second 6 months post‐HSCT and every 3 months in the second year. Most stop monitoring at 2 years post‐HSCT.

In AML, any mixed chimerism post‐HSCT is a concern.^28^ Early withdrawal of IS with or without DLI is common practice. There is an increasing trend, in the United Kingdom, to use umbilical cord blood transplantation (UCBT) without serotherapy in high‐risk AML. Compared with other cell sources, T cell‐replete cord blood transplant results in improved disease‐free survival and relapse risk in paediatric AML/MDS and the incidence of mixed chimerism is low after such transplants,^40^ DLI is usually not an option. The same schedule of chimerism monitoring is used as in ALL and co‐relation with MRD remains a vital component of optimal care.

Juvenile myelomonocytic leukaemia (JMML) is a rare and aggressive myeloproliferative neoplasm of childhood. Nearly all patients have a mutation detected in the RAS pathway, and the majority need HSCT with a myeloablative conditioning regimen. The role of pretransplant chemotherapy is debatable, and many children will not be in a CR at HSCT. There is a very high incidence of relapse post‐transplant (nearly 50%). It is recognised that GVL plays a very strong role in cure and early withdrawal of IS is crucial.^41^ BMA every 90 days for the first 18 months, when most relapses occur, has been suggested, although this is not performed routinely in the United Kingdom.^42^ In some institutions, there is monitoring of PB lineage‐specific chimerism every 2 weeks until off IS. Monocyte (CD14+) chimerism is checked in the event of mixed chimerism in whole (unfractionated) PB samples. There is a role for post‐transplant DLI ± azacitidine, although most relapses occur aggressively and early after BMT, which does not allow time to engineer immunomodulation.^43^ The success of DLI is variable and is often not effective as may be predicted for this disease. For this reason, and because a second BMT can often salvage patients with JMML, there is an argument for not administering DLI and proceeding to a second transplant if the child is fit.^43^, ^44^

#### Non‐malignant disease indications

This encompasses inborn errors of immunity, acquired bone marrow failure (BMF), inherited BMF syndromes, metabolic disorders and haemoglobinopathies. Lineage‐specific chimerism monitoring is mandatory. Different diseases require different levels of chimerism in the diseased lineage (Table 4).^45^ Clinicians should have an awareness of when mixed chimerism is of no consequence. In different diseases, other functional testing may be used alongside the results of chimerism to determine the significance of MC, for example, dihydrorhodamine (DHR test) in chronic Granulomatous Disease or enzyme levels in Hurler's syndrome.

**TABLE 4:** Lineage‐specific chimerism requirements and necessary levels of donor chimerism for successful transplant outcomes in non‐malignant disorders.

Non‐malignant disorder	Lineage specificity	Minimal level of donor chimerism for cure
Inherited immunodeficiencies		
HLH	NK/T cell	>30%
SCID	T/B/NK	T cell −100%
CGD	Myeloid	>50%
WAS	Lymphoid/myeloid	>50%
Haemoglobinopathies		
Sickle cell disorders	Erythroid/myeloid	>20%–25%
Thalassaemia	Erythroid/myeloid	>20%–25%
Metabolic diseases		
ALD, Hurler's	Myeloid	70%–100%
Osteopetrosis	Myeloid	>10%
Bone marrow failure syndromes		
SCN, DBA, FA, SDS	Myeloid	100%
	Lymphoid	>50%

*Note*: From Zimmerman and Shenoy.^45^

#### Acquired aplastic anaemia

Clinical outcomes are excellent using alemtuzumab‐containing regimens, and MC in T‐cell lineage is very common. Recipient‐derived CD8 T cells shape persistent T‐cell MC^3133^ and this is of no consequence to disease cure. A high level of myeloid engraftment is desirable. Attempts should not be made to improve T‐cell chimerism by reducing IS as any significant GVHD would be deleterious to patient outcome.

#### Inborn errors of immunity

Intensity of conditioning depends on the underlying disorder. The choice of conditioning regimen is an evolving field as novel genetic defects are discovered.^46^ Lineage‐specific chimerism is crucial and full donor chimerism is not needed for disease cure. There are differing levels of desired chimerism in different diseases and low‐level chimerism in the non‐disease lineage is acceptable. Partial disease correction may occur in the presence of mixed chimerism.

#### BM failure syndromes

Most require RIC depending on the underlying disorder. Full donor chimerism (FDC) in myeloid lineage is desirable due to the risk of future myeloid malignancies. Mixed chimerism in lymphoid lineage is acceptable but long‐term follow‐up is essential to evaluate the risk of clonal haematopoiesis.

#### Haemoglobinopathies

Haemoglobinopathies are an increasingly common indication for HSCT in children and adults. Mixed chimerism is common even with myeloablative conditioning. Myeloid lineage chimerism is a good surrogate for erythropoiesis in the absence of red cell chimerism (CD71) evaluation. Younger age is the main factor in determining mixed chimerism independent of the degree of matching and is a risk for rejection.^47^, ^48^ Graft rejection is not related to the time of onset of mixed chimerism, but to a donor fraction of <90% in whole blood; strategies to address lymphoid engraftment are successful in reversing the risk.^49^, ^50^, ^51^ As seen in the adult population, unstable mixed chimerism can lead to clonal evolution.^52^ Therefore, assessment of whole blood and T‐cell fraction is required at day +28, +60, +100 and continued according to results until cessation of IS. If chimerism drops below 90%, there should be an assessment of BM cellularity and chimerism, and if interventions are instituted, surveillance may be increased to 2 weekly. Overall, there is an acceptance of stable mixed chimerism, but there is a need to monitor this for at least 2–3 years as a quarter of all rejections occur >1 year post‐transplantation.^53^

The current UK practice for chimerism monitoring in other non‐malignant disorders includes WB chimerism at engraftment, with lineage‐specific chimerism if any evidence of MC except in haemoglobinopathies where lineage‐specific chimerism monitoring is necessary. Monthly chimerism for the first 6 months; 2–3 monthly until a year post‐HSCT; 3 monthly in the second year; and then annually to 5 years.

- **Disease‐specific schedules for chimerism testing of unfractionated or specific lineage samples are often indicated in both malignant and non‐malignant conditions in children.** ^2^, ^3^
- **Clinicians should be aware that individual non‐malignant conditions require disease‐specific levels of chimerism in the affected lineage and know when mixed chimerism is of no consequence.** ^2^, ^3^
- **In haemoglobinopathies, assessment of whole blood and lineage‐specific chimerism in, for example, T‐cell fraction (CD3), myeloid fraction (CD15) at day +28, +60, +100 and at least 3 monthly to cessation of immunosuppression is standard of care to guide the management of immunosuppression.** ^1^, ^2^
- **For patients undergoing transplant for non‐malignant conditions (predominantly in children) where mixed chimerism is observed long‐term, follow‐up to 5 years is needed to assess for clonal evolution or other disease manifestations.** ^1^, ^2^
- **In acute leukaemia in children, whole blood chimerism is recommended monthly in the first 6 months, 2 monthly to 1 year and 3 monthly in the second year. Lineage‐specific (T and myeloid) chimerism should be checked if there is any evidence of mixed unfractionated chimerism.** ^2^, ^3^
- **In children with leukaemia, MRD should be correlated with chimerism results to guide treatment decisions.** ^2^, ^3^
- **In JMML fortnightly whole blood and CD14 lineage‐specific chimerism is a clinical option to help optimise GvL.** ^2^, ^3^

## DISCUSSION

There are currently significant variations in chimerism testing across the UK community at both clinical and laboratory levels. While some of this variation may relate to specialist transplant practice, other aspects may be more a historical ‘convention’ that centres and their associated laboratories have arrived at over the years. The lack of robust prospective evidence potentially explains why centres arrive at their own policies through local expertise, experience and financial resources. In recent years, however, there have also been changes in the NHS provision of laboratory testing with greater centralisation of molecular genetic services, which has affected the provision of chimerism testing in many UK transplant centres. There is a need to ensure this testing reflects clinical priorities.

Although there have been some previous laboratory (EQA‐based) recommendations,^7^ there have never been published guidelines from the UK clinical transplant community on chimerism testing. Internationally, in recent years, other than some broad guidance from EBMT, there has been relatively little published aimed at harmonisation of best practice in this area. We have attempted to address some of these important questions using the approaches discussed.

In terms of laboratory testing, we were able to generate recommendations with a high degree of consensus. There was agreement regarding TATs and the need to report both sensitivity and purity values on laboratory reports. Support for participation in EQA programmes was 100%. The use of STR‐based testing is standard practice in the United Kingdom and it is informative, rapid, reliable, accurate and reproducible, despite limited sensitivity of 1%–5%, with almost all the predictive value of mixed chimerism on transplant outcomes and the thresholds used in studies of intervention using chimerism as a trigger for DLI being based on this technology. Newer techniques, for example, q‐PCR, dPCR or NGS, are more sensitive and detect the lower levels of residual or recurrent host haemopoiesis, termed microchimerism, but unanswered questions remain in relation to clinical relapse rates and survival and the levels of chimerism triggering interventions in various clinical contexts.

Arriving at consensus to guide clinical practice is more challenging, especially in malignant diseases, because using mixed chimerism alone to trigger intervention with DLI remains contentious. Simply detecting recipient cells in an individual patient does not necessarily mean the patient will relapse if left untreated. As a result, it remains unknown when, if at all, individual patients treated pre‐emptively would have relapsed had they not been given DLI infusions, leading to both lead time and selection biases in the published literature.^54^ Conversely, the proven activity of DLI in strengthening the GvL effect is undoubted, and clinicians believe to varying degrees that there is a place for such interventions, explaining variability in clinical practice between centres and on an individual patient basis.

These problems were particularly pronounced in adult clinical practice, where transplants are more commonly performed for malignant disease using reduced intensity T‐depleted conditioning and necessitating therapeutic decisions based on chimerism testing as triggers for tapering of IS and ‘pre‐DLI’. We were able to gain consensus to recommend whole blood chimerism (unfractionated PB/BM) being performed at day +30 using STR technology for malignant diseases, with persistent or rapidly worsening mixed chimerism <95% donor in either whole blood or T‐cell fractions, requiring intervention with tapering of IS ± DLI. However, individual decisions should be made depending on the clinical context. In paediatric practice, there is more widespread consensus. DLI are used less frequently given and other options such as CAR‐T are used more often. Practical thresholds for intervention do exist in many diseases, including in non‐malignant conditions testing schedules (Table 4). In both adult and paediatric practice, chimerism analysis and MRD monitoring are important complementary monitoring modalities post allogeneic stem cell transplant and should be monitored in tandem.

In 2016, the EBMT reviewed methods of monitoring for relapse in AML post‐BMT with specific emphasis on MRD and chimerism, and our UK recommendations broadly agree.^55^, ^56^ They were not proscriptive in terms of timings or thresholds and have suggested that chimeric abnormalities should be interpreted in the context of the patients' clinical and laboratory results without providing detailed guidance for intervention. The EBMT recommendations have subsequently been updated in 2024 with more general disease applicability, including proposals for starting doses of DLI and criteria for safe delivery of these cells, giving general support for the role of ‘preDLI’ in the context of mixed chimerism, as well as for persistent or emergent MRD but without specified threshold values of mixed chimerism or mandatory testing strategy.^57^ They do highlight the need to standardise clinical testing schedules, laboratory procedures and data registry reporting to promote a fuller understanding of and harmonise current practices. We have, therefore, recommended that the EBMT guidelines are routinely consulted and included in institutional protocols.

In summary, the multidisciplinary meeting and consultation exercise brought together clinical and laboratory expertise to appraise and develop UK NHS practice and collaborative working. BSBMTCT aims to maintain this network to exchange information, facilitate research and data collection in a coordinated manner in association with novel technologies and evolving therapeutic strategies.

## AUTHOR CONTRIBUTIONS

All authors contributed to the writing of this manuscript. AC, HC, KR, NF, JDF, JL, TM, JS wrote sections within the first draft of the paper. AH, EO, DR, PT, VP reviewed and contributed substantial contributions to the final manuscript.

## List of attendees

**Table:**

Dr.	Adele Timbs	Oxford University NHS Foundation Trust
Professor	Adrian Bloor	The Christie NHS Foundation Trust
ms	Agie Fogarty	CHI Crumlin
Dr	Ailsa Holroyd	Queen Elizabeth University Hospital
Dr	Andrew Clark	Queen Elizabeth University Hospital, Glasgow
Dr	Andy Peniket	Oxford University Hospitals
Dr	Angela Hamblin	Oxford University Hospitals NHS Foundation Trust
Mr	Angus Haines	The Royal Marsden
Dr	Anjum Khan	St James' Hospital Leeds
Dr	Anna De Palma	Cambridge University Hospitals
Dr	Anoop Cherungonath	Birmingham children's hospital
Dr	Anthony Poles	NHSBT—Bristol
Dr	Antony Cousins	Sheffield Children's Hospital
Dr	Arthi Anand	Imperial College NHS trust
Mrs	Charlotte uddin	NUH NHS trust
MR	David Fishwick	UHS NHS FT
Dr	David Irvine	QEUH GGC Scotland
Dr	Deborah Richardson	University Hospital Southampton
Dr	Deborah Sage	NHS Blood and Transplant
Dr	Deniz Ucanok	Nottingham University Hospital
Dr	Dimitris Galopoulos	Queen Elizabeth University Hospital
Dr	Dorte Wren	Great Ormond Street Hospital/NT‐GLH
Professor	Eduardo Olavarria	Imperial College—Hammersmith Hospital
Dr	Emma Nicholson	RMH
Dr	Erin Hurst	Newcastle upon Tyne NHS Foundation Trust
Dr	Fotini Partheniou	Royal Liverpool Hospital—LUHFT
Dr	Grant McQuaker	Queen Elizabeth University Hospital, Glasgow
Dr	Harpreet Kaur	Sheffield Teaching Hospitals NHSFT
Dr	Hazel Clouston	UK NEQAS LI
Dr	Helena Lee	Manchester Royal Infirmary
Dr	Jennifer Clay	Leeds teaching hospitals NHS trust
Dr	Jennifer Edwards	Nottingham University Hospitals
Mrs	Jennifer Jones	CUH—Addenbrookes Hospital
MIss	Jennifer Stevens	UHS
Ms	Jianne Miran	King's College Hospital
Ms	Joanne Mason	Birmingham
PROF	JOHN SNOWDEN	Sheffield Hospitals NHS Trust
Professor	Josu de la Fuente	Imperial College Healthcare NHS Trust
Dr	Justin Loke	University of Birmingham
Dr	Kanchan Rao	GOSH
Mrs	Kathryn Turner	Leeds Teaching Hospitals Trust
Dr	Katy Latham	NHSBT
Mr	King Hei Lai	University Hospital Southampton NHS
Dr	Kirsty Sharplin	Oxford University Hospital
Miss	Lauryn Wills	Synnovis
Dr	Leigh Keen	NHSBT—Filton
Ms	Lorna welsh	Queen Elizabeth University Hospital
Miss	Maia Hickin	The Royal Marsden
Dr	Manoj Raghavan	University Hospitals Birmingham
Mrs	Maria Azucena Losa	Queen Elizabeth Hospital, Birmingham
Mrs	Marianne Grantham	Barts Health NHS Trust
Miss	Megan Mitchell	The Royal Marsden
Mrs	Michele Barrett	NHSGGC
Ms	Michelle Kenyon	King's College Hospital NHS Foundation Trust
Dr	Najeem Folarin	King's College Hospital, Synnovis
Mrs	Nancy Atieno	University Hospital Southampton
Dr	Natalia Brodaczewska	Imperial College Healthcare NHS Trust
Ms	Nicola Meakin	University Hospital Southampton
Dr.	Noora Buti	Imperial college NHS trust
Dr	Oluwatosin Taiwo	The Royal Marsden NHS Foundation Trust
Dr	Pamela Evans	CHI at Crumlin
Dr	Patrick Medd	University Hospitals Plymouth NHS Trust
Mrs	Polly Talley	St James, Hospital, Leeds
Dr	Prudence Hardefeldt	Kings College Hospital
Dr	Renuka Palanicawandar	Hammersmith Hospital
Dr	SAMAH LAMBURNE	Southampton University Hospital
Dr	Sandeep Potluri	Birmingham Children's Hospital
Mrs	Sanna Hulkki Wilson	The Royal Marsden Hospital
Mrs	Sarah Darko	Oxford University Hospitals NHS Foundation Trust
Dr	Sarah Lawson	Birmingham Children's Hospital
Dr	Sharon Vivers	Anthony Nolan
Dr	Shaun Bevan	Barts Health
Professor	Stuart Adams	Great Ormond Street Hospital for Children NHS Foundation Trust
Miss	Susanne Kricke	Great Ormond Street Hospital
Ms	Tasmiya Wahed	Kings College London
Dr	Terri McVeigh	Royal Marsden
Dr	Valerie Broderick	CHI at Crumlin
Dr	Wendy Ingram	University Hospital of Wales
